# Vascular cognitive impairment: When memory loss is not the biggest challenge

**DOI:** 10.1177/14713012231214299

**Published:** 2023-11-24

**Authors:** Sara AJ van de Schraaf, Merel F Smit, Majon Muller, Cees MPM Hertogh, Hanneke FM Rhodius-Meester, Eefje M Sizoo

**Affiliations:** Amsterdam UMC, Location Vrije Universiteit Amsterdam, Medicine for Older People, Amsterdam, Netherlands; 1229 Amsterdam Public Health, Aging & Later Life, Amsterdam, Netherlands; 1209 Amsterdam UMC, Location Vrije Universiteit Amsterdam, Internal Medicine, Geriatric Medicine Section, Amsterdam, Netherlands; Amsterdam UMC, Location Vrije Universiteit Amsterdam, Medicine for Older People, De Boelelaan 1117, Amsterdam, Netherlands; 1229 Amsterdam Public Health, Aging & Later Life, Amsterdam, Netherlands; Amsterdam UMC, Location Vrije Universiteit Amsterdam, Internal Medicine, Geriatric Medicine Section, De Boelelaan 1117, Amsterdam, Netherlands; 171890 Amsterdam Cardiovascular Sciences, Atherosclerosis & Ischemic Syndromes, Amsterdam, Netherlands; Amsterdam UMC, Location Vrije Universiteit Amsterdam, Medicine for Older People, De Boelelaan 1117, Amsterdam, Netherlands; 1229 Amsterdam Public Health, Aging & Later Life, Amsterdam, Netherlands; Amsterdam UMC, Location Vrije Universiteit Amsterdam, Internal Medicine, Geriatric Medicine Section, De Boelelaan 1117, Amsterdam, Netherlands; Amsterdam Cardiovascular Sciences, Atherosclerosis & Ischemic Syndromes, Amsterdam, Netherlands; Amsterdam UMC, Location Vrije Universiteit Amsterdam, Alzheimer Center Amsterdam, Neurology, De Boelelaan 1117, Amsterdam, Netherlands; Amsterdam Neuroscience, Neurodegeneration, Amsterdam, Netherlands; Oslo University Hospital, Geriatric Memory Clinic, Kirkeveien 166, Oslo, Norway; Amsterdam UMC, Location Vrije Universiteit Amsterdam, Medicine for Older People, De Boelelaan 1117, Amsterdam, Netherlands; 1229 Amsterdam Public Health, Aging & Later Life, Amsterdam, Netherlands

**Keywords:** vascular dementia, cognitive disorder, dementia, long-term care, family care, dementia care

## Abstract

**Objectives:**

Vascular cognitive impairment is the second most common type of cognitive impairment. Care needs of community-dwelling people with vascular cognitive impairment and their caregivers have not been thoroughly studied. Therefore, we aimed to explore care needs of people with vascular cognitive impairment and their family caregivers.

**Design:**

A qualitative interview study.

**Setting and participants:**

Participants were purposefully sampled community-dwelling people with vascular cognitive impairment and their family caregivers.

**Methods:**

Interviews were audiotaped and transcribed verbatim. Analysis and data collection followed an iterative process, until data saturation was achieved. We conducted 18 interviews (nine people with vascular cognitive impairment and nine caregivers), concerning 13 unique people with vascular cognitive impairment. We analyzed the data using inductive thematic analysis following the Braun & Clark method. The study was reported in accordance with the COREQ criteria.

**Findings:**

Five themes were identified in the care needs reported by people with vascular cognitive impairment and family caregivers: (1) Specific information need with subtheme (1A) No memory problem, no dementia? (2) Being respected as a person, (3) Differing concerns about the future, (4) The roles of the caregiver and (5) Decisiveness from professional healthcare.

**Conclusions and implications:**

The care needs of people with vascular cognitive impairment and their caregivers were affected by (a lack of knowledge about) the characteristic symptoms of this condition. Participants equated cognitive impairment or dementia to memory loss (“Alzheimerization”), although memory loss was not their biggest challenge. People with vascular cognitive impairment and caregivers preferred resolute and decisive healthcare professionals. These professionals activate the person with vascular cognitive impairment who lacks initiative and diminishe role conflict of the caregiver. Care for people with vascular cognitive impairment and their caregivers could be improved by providing tailored information, promoting awareness of neuropsychiatric symptoms, particularly apathy, and by healthcare professionals providing more guidance in decision-making.

## Introduction

The number of people affected by cognitive impairment and dementia worldwide continues to rise (Nichols et al., 2022), which impacts people living with the condition, their family caregivers and healthcare professionals (Hugo & Ganguli, 2014). Increasing prevalence will increase the already significant burden on family caregivers (Etters et al., 2008) and the need for optimal personalized care. Several studies have investigated care needs of people with cognitive impairment and family caregivers, highlighting unmet needs in multiple dimensions of care, such as information provision and professional guidance (Beunder et al., 2015; Boots et al., 2015; Dam et al., 2018; Granbo et al., 2019; Lindblom et al., 2020; Stiekema et al., 2020; van der Roest et al., 2007, 2009; Wolfs et al., 2010). (For further information, see the Background section). Unmet needs of people with dementia and their caregivers are associated with earlier nursing home admissions, increased mortality and depressive symptoms (Black et al., 2013; Gaugler et al., 2005). Notably, studies have indicated more unmet needs and lower capacity to ‘live well’ among people with cognitive impairment due to other causes than Alzheimer’s disease (van der Roest et al., 2009; Wu et al., 2018).

The second most common cause of cognitive impairment is vascular disease (Dichgans & Leys, 2017). The term vascular cognitive impairment is used to describe all types of cognitive impairment associated with cerebrovascular disease (see Box 1). Symptoms of vascular cognitive impairment are diverse and partly overlap with other causes of cognitive impairment (Dichgans & Leys, 2017; van der Flier et al., 2018). The specific symptoms associated with vascular pathology, such as psychomotor slowing and apathy, as well as the non-linear disease course, could have important implications for focus of care (Bruandet et al., 2009; Schwertner et al., 2022; van de Schraaf et al., 2022).

**Box 1: What is vascular cognitive impairment?**

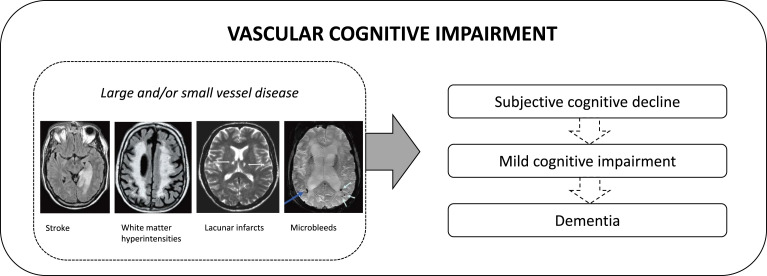

Vascular cognitive impairment (Dichgans & Leys, 2017; van der Flier et al., 2018) is a relatively new term that resembles the continuum of cognitive disorder from mild cognitive changes to dementia, associated with cerebrovascular disease. This includes diagnoses such as mild cognitive impairment due to cerebrovascular pathology, cognitive impairment after stroke and vascular dementia. The term vascular cognitive impairment was introduced around 20 years ago to “[broaden] the current narrow definitions of vascular dementia […] to recognise the important part cerebrovascular disease plays in several cognitive disorders”(O’Brien et al., 2003). Pathology of vascular cognitive impairment includes large-vessel disease, such as stroke, and small-vessel disease leading white matter hyperintensities, lacunar infarcts and microbleeds. Vascular pathology is the second most common cause of dementia, after Alzheimer’s disease, accounting for at least 15–20% of dementia cases (Goodman et al., 2017; Gorelick et al., 2011). The prevalence of mild cognitive impairment due to vascular pathology is estimated to be even higher (Rockwood et al., 2000). Vascular cognitive impairment frequently co-exists with neurodegenerative pathology, leading to mixed disease or dementia.

Despite vascular cognitive impairment being a common type of cognitive impairment and dementia, research on care needs of people with vascular cognitive impairment or vascular dementia and their family caregivers is very limited. One retrospective medical record study (Jhang et al., 2020) stressed the importance of caregiver mood. We believe a more comprehensive approach is needed to identify experienced care needs from people with vascular cognitive impairment and caregivers. A qualitative approach permits exploring topics important to people with vascular cognitive impairment and caregivers, rather than topics determined by researchers and policymakers. Therefore, we conducted a qualitative study to explore care needs of people with vascular cognitive impairment and their caregivers.

## Background

Care needs have been studied in several populations comparable to people with vascular cognitive impairment, such as people with dementia, Alzheimer’s disease and stroke. In this section, we will elaborate on the earlier literature on care needs in these populations.

Studies on care needs of people with dementia and their caregivers have predominantly focused on or included people with Alzheimer’s disease. Needs of people with dementia often revolve around their ability to accept and cope with the condition. They come to terms with their new situation, while seeking to be treated as equals by the people around them (van der Roest et al., 2007). However, people with dementia do not report explicit ideas on how to fulfil those needs. Both people with dementia and caregivers want more information about the condition, available treatments and care services. People with dementia and caregivers are often unaware of treatment and care options, which can result in suboptimal utilization of available care (Boots et al., 2015; Dam et al., 2018; Granbo et al., 2019; van der Roest et al., 2009; Wolfs et al., 2010). People with dementia and caregivers report that increased communication with healthcare professionals would improve their awareness of available care and increase their empowerment (Dam et al., 2018; Wolfs et al., 2010).

In stroke literature, several studies have investigated the needs of people with stroke after the acute phase and during rehabilitation, or when returning home (Beunder et al., 2015; Lindblom et al., 2020; Stiekema et al., 2020). In these studies, participants have emphasized the lack of adequate care and support in the home environment, particularly concerning cognitive impairment. People with stroke and caregivers experience discharge from the hospital as sudden, feeling uninformed, unprepared and uncertain about the future. They feel fully responsible for their own recovery and care trajectory, yet they lack the knowledge to take on this responsibility. (Beunder et al., 2015; Lindblom et al., 2020; Stiekema et al., 2020). In addition, cognitive ‘hidden’ consequences of stroke become more apparent in the long term, in the months or years following the event, when individuals attempt to fully resume their daily routines. However, many do not receive formal care during this phase (Beunder et al., 2015; Stiekema et al., 2020). People with stroke and caregivers wish for more information (for themselves, society at large and some healthcare professionals), more long-term guidance and more attention for cognitive and emotional consequences of stroke (Beunder et al., 2015; Lindblom et al., 2020; Stiekema et al., 2020).

Caregivers of people with stroke and caregivers of people with dementia face several similar challenges. Caregivers report feeling burdened as a result of the diminished functioning of their relative or while struggling to adapt to the new situation (Boots et al., 2015; Granbo et al., 2019; Stiekema et al., 2020; van der Roest et al., 2009). Caregivers highlight how their relative’s condition changed their interpersonal relationship. Consequently, they experience role conflicts and stress their need for personal time (Boots et al., 2015; Granbo et al., 2019; Lindblom et al., 2020; Stiekema et al., 2020).

## Methods

### Participants and sampling

We approached community-dwelling people with vascular cognitive impairment who had received a diagnostic workup at the geriatric memory clinic of the Amsterdam UMC (see Table 1 for inclusion- and exclusion criteria). We purposefully sampled individuals that could provide in-depth and complementary information, to ensure sufficient variety in the data (Palinkas et al., 2015). We sampled on age, sex, education level, marital status, severity of cognitive impairment, time since diagnosis, types of cerebrovascular damage and types of care received. We based these criteria on important characteristics in previous research (Dam et al., 2018; Wolfs et al., 2010) and iteratively updated these to include variant cases. For example, we included ‘care received’ as a purposeful sampling category when we noticed that we had not yet included participants who received homecare.

**Table 1.** Inclusion and exclusion criteria of the study.

Inclusion criteria	
1)	Presence of mild cognitive impairment or dementia.^a^
2)	Significant cerebrovascular damage on brain imaging, adapted from the NINDS-AIREN criteria (Román et al., 1993), at least one of the following
	- Presence of large confluent areas of deep white matter hyperintensities (fazekas score = 3)(Fazekas et al., 1987)
	- Large-vessel infarctions
	- Strategic lacunes in the basal ganglia or thalamus
3)	Sufficient proficiency of the Dutch language
Exclusion criteria
1)	Mixed etiology of cognitive impairment in which other etiologies – such as Alzheimer’s disease or frontotemporal dementia - were predominant
2)	For interviews with people with vascular cognitive impairment: Institutionalization prior to the interview

*Note.*

^a^One person with subjective cognitive decline was interviewed for purposive sampling.

SvdS and MS approached eligible people with vascular cognitive impairment by sending an information letter and making a follow-up telephone call one week later. We contacted nineteen people with vascular cognitive impairment in three rounds (*n* = 10, *n* = 6, *n* = 3). After each round, we discussed which subjects to approach next, taking into account the current variety in our purposeful sample. This was followed by checking if saturation had been reached (see Procedure). The caregiver was contacted with permission of the person with vascular cognitive impairment. Of the contacted people with vascular cognitive impairment, in thirteen cases the person with vascular cognitive impairment and/or caregiver participated (see Supplement 1 for non-participation reasons). In total, eighteen participants (nine people with vascular cognitive impairment and nine informal caregivers) were interviewed (see Table 2 for participant characteristics).

**Table 2.** Background characteristics of the participants.

	People with vascular cognitive impairment (*n* = 9)											Caregivers (*n* = 9)			
	Age	Sex	Education level^a^	Deep WMH: Fazekas score	Lacunar stroke (*n*)	CVA, large vessel (yes/no)	MMSE	MoCA	CDR	Marital status		Relation to person with vascular cognitive impairment	Age	Sex	Education level^a^
P7^b^	81	M	Middle	2	2^c^	Yes	27	24	0.5	Married	C7	Partner	61	V	University
P12	75	M	Middle	2	0	Yes	26	22	0.5	Married					
^d^	*71*	*M*	*University*	*3*	*3*	*Yes*	*24*	*16*	*1*	*Married*	C13	Partner	70	V	Higher
P15	85	M	Middle	3	1	No	22	15	0.5	Living together	C15	Partner	68	V	Lower
P20	76	F	Middle	3	3	No	28	26	0.5	Widowed	C20	Daughter-in-law	52	V	Higher
P24	75	M	Lower	2	1^c^	Yes	26	22	1	Married	C24	Partner	71	V	Lower
P26	63	F	Middle	2	4^c^	No	27	24	0.5	Divorced					
^d^	*81*	*F*	*Lower*	*3*	*0*	*Yes*	*19*	*12*	*2*	*Married*	C29	Partner	76	M	Higher
^d^	*77*	*M*	*Higher*	*3*	*1* c	*No*	*27*	*20*	*1*	*Married*	C33	Partner	76	V	Lower
P34	88	F	University	3	0	No	26	22	0	Widowed	C34	Son	62	M	University
P41	91	F	Lower	3	2^c^	No	22	18	1	Divorced					
P42	69	M	University	1	1^c^	No	30	23	1	Single					
^d^	*78*	*M*	*University*	*3*	*0*	*Yes*	*22*	*19*	*1*	*Widowed*	C45	Daughter-in-law	52	V	University

^a^Education level: lower = primary education/lower preparatory vocational education/lower vocational education, middle = higher preparatory vocational education/secondary vocational education, higher = senior general/pre-university secondary education and higher vocational education.

^b^People with vascular cognitive impairment and caregivers with corresponding numbers are from the same dyad.

^c^Strategic lacunar infarction in the basal ganglia or thalamus.

^d^People with vascular cognitive impairment whose caregiver participated, but who did not participate themselves.

Note. M: Male; F: Female; WMH: White Matter Hyperintensities; CVA: Cerebrovascular Accident; MMSE: Mini Mental State Examination; MoCA: Montreal Cognitive Assessment; CDR: Clinical Dementia Rating.

All participants provided informed consent. The Medical Ethics Committee of the Amsterdam UMC, location VUmc approved the study protocol prior to subject inclusion (2020.0746).

### Procedure

Interviews were conducted between February and July 2021. Sampling, data collection and analysis were performed in an iterative and reflexive process, meaning that these steps of the process occur simultaneously and in a back-and-forth manner. Consequently, insights that occurred during the process shaped further data collection and analysis (Srivastava & Hopwood, 2009). Data collection ended when we reached ‘saturation’: when we perceived richness and thickness in data, and topics discussed in later interviews fitted existing codes and themes (Saunders et al., 2018; Sebele-Mpofu, 2020). We expected saturation after the first fifteen interviews (two rounds), and this was confirmed with three additional interviews.

Semi-structured interviews were conducted by SvdS and MS. Each interviewer conducted one pilot interview, of which one was not included in the final analysis because of technical difficulties. Interviews were conducted through video call or telephone, depending on the participant’s preference. Live interviews were not possible due to the COVID-19 regulations. All participants were asked to be alone during the interview, but we allowed caregivers to be present if this made the person with vascular cognitive impairment more comfortable. Four included people with vascular cognitive impairment had had previous contact with SvdS for neuropsychological evaluation: these participants were interviewed by MS to avoid bias.

The interviews were guided by a topic list, generally referring to the participants experience of received and desired care. After the first few interviews, this topic list was revised to create two separate topic lists, because more differentiation was needed to relate to the lived experiences of the people with vascular cognitive impairment versus the caregivers (Supplement 2). All interviews were audiotaped and transcribed verbatim. Identifiable information was not included in the transcript. Field notes were made during and after every interview. To improve the validity of the study, we performed a member check; all respondents received a summary of the interview to review. Two participants provided comments regarding information not included in the summary.

### Data analysis

We analyzed the data using inductive thematic analysis according to the approach by Braun and Clarke (2006). We aimed to study the lived experiences of the participants (phenomenological methodology) (Giorgi, 1997). Coding and analysis were performed in the software programs Atlas.ti 9 and MAXQDA 2020.

Before coding, the authors read the transcripts thoroughly, summarized the transcripts and registered remarkable observations (step 1 of thematic analysis according to Braun and Clarke (2006)). All interviews were coded independently by two researchers (Step 2); SvdS and MS coded the first seven interviews, while SvdS and ES coded later interviews. Through axial and iterative coding, codes were grouped into higher-order codes, categories and themes (Step 3). After initial analyses, themes were discussed in meetings with the whole research group and refined according to feedback received (Step 4, Supplement 3). After reflexive analysis and reaching saturation, final themes and theme names were approved by all co-authors (Step 5). This manuscript was written (Step 6) in accordance with the COREQ checklist for qualitative research (Supplement 1) (Tong et al., 2007).

## Findings

**Figure 1.**
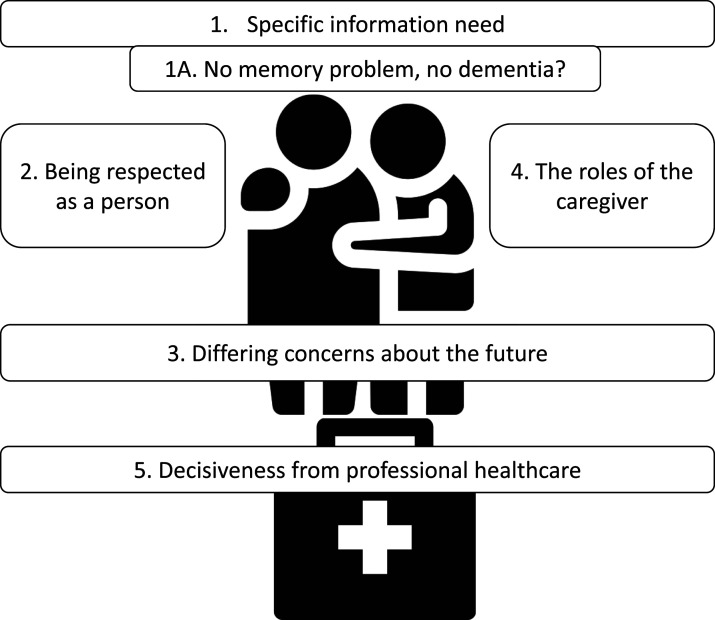
Themes in the care needs reported by people with vascular cognitive impairment and family caregivers.

We identified five themes through inductive analysis (Figure 1; coding cloud provided in Supplement 3). The responses of people with vascular cognitive impairment and caregivers are handled separately within the themes, as their answers differed in focus and opinions related to the themes. Interview duration differed substantially between interviews with people with vascular cognitive impairment (28–61 minutes) and caregivers (59–86 minutes). This reflected the caregivers’ tendency to spontaneously elaborate on their answers to the questions asked by the interviewers.

### Theme 1: Specific information need

This theme encompasses the need for information about vascular cognitive impairment and the subsequent mental representation of what the condition entails. Some participants were unaware that mild cognitive impairment due to vascular pathology or vascular dementia was the primary diagnosis. Often, they did recall whether the diagnosis contained the term dementia.

“Then we did the examination and fortunately it showed that it wasn’t actual dementia yet” (Caregiver in Interview P12)

People with vascular cognitive impairment did not express a desire to educate themselves on their condition or its consequences.

“Well, I don’t need any more information about anything. No.” (P15)

Caregivers reported a higher need for information. They expressed that healthcare professionals did not always provide sufficient information. Therefore, caregivers gathered their information from other sources, like websites and (scientific) articles. Based on their own experiences, they reported a mismatch between the diagnostic terminology used by the memory clinic and the severity of the symptoms.

“Mild cognitive impair— Yes, I must say, that I believe... that I sometimes really think, like, well, I believe he is really very bad sometimes. And I think, well, I don’t know, but if this is mild cognitive impairment, well then, I don’t think so. It seems to me that it's a bit worse than that.” (C7)

Caregivers were especially interested in information on the relationship between the vascular damage and the changes in behavior or functioning of the person with vascular cognitive impairment.

“What is found and considered to be normal, including the [white matter] abnormalities that come with age, how normal is that compared to others and how do others respond to that? Like, what are the expectations more or less?” (C34)

On top of that, caregivers perceived not all healthcare professionals had adequate knowledge of vascular cognitive impairment. They felt that enhanced knowledge of healthcare professionals would result in better understanding and improved management of the behavior of the person with vascular cognitive impairment.

“That she has had a stroke in the past. That your character is… that it changes it so much. […] I believe the nurses don’t always understand that. I think they aren’t sufficiently aware of that.” (C29)

#### Subtheme 1A: No memory problem, no dementia?

The way participants talked about the cognitive diagnosis followed a certain pattern. They tended to describe the diagnosis mostly in relation to memory deficits or Alzheimer’s disease.

“They said at the time that it wasn’t Alzheimer’s, so to speak, but that I might have a predisposition to Alzheimer’s anyway.” (P26)

“I didn’t immediately suspect that it had something to do with dementia. […] He has no memory loss.” (C13)

Caregivers who had gathered information on vascular cognitive impairment stressed the importance of distinguishing between different causes of cognitive impairment.

“What does that mean, what can you expect? Very important. Yes, because it’s clearly someone with vascular [dementia]. Because someone with Alzheimer’s… they’re quite different in their behavior towards others. And if you don't know that, how can you recognize it? […] That’s made a difference… that at a certain point I thought: yes, there’s nothing I can do. It’s something in your brain” (C33)

### Theme 2: Being respected as a person

One of the core wishes of the people with vascular cognitive impairment was to be treated with value and respect: not as a patient, but as a person. An important element of being treated as a person was being valued by others.

“So, the feeling of things being usual, relaxed, safe and being acknowledged. And preferably that they are happy with you, because that makes for even more fun [in life]” (P20)

“I can be of value and everyone is equal there [at the day care center]. So, whether I have dementia or not, I’m not the only one with a problem. So, I feel very comfortable there, so to speak.” (P7)

People with vascular cognitive impairment did not express many explicit care needs. Instead, they emphasized wanting to continue their lives similarly to pre-diagnosis, as an autonomous individual. Their wish was to continue activities and hobbies and remain as independent as possible. Even so, they admitted that remaining completely independent was not always possible.

“I try to do as many things myself as possible. But it doesn’t always work out with everything.” (P12)

“I hate a dirty house, believe me, I loathe it. Then I’ll clean it. But my cleaning isn’t like it used to be.” (P41)

People with vascular cognitive impairment were concerned with losing their independence, which was especially apparent when discussing losing their driver’s license and car.

“I’m not allowed to drive anymore! That’s really bad […]. And so, I need support, because someone has to drive me there.” (P42)

Caregivers repeated the statement that their loved ones would prefer not to be addressed as patients.

“My mother’s desire is precisely that she wants to go back to normal. […] So I just want to get rid of the image that people have of her as a patient… being a patient forever and ever.” (C34)

On top of that, caregivers expressed that it was important to them that their loved ones were being taken seriously. They wanted the person with vascular cognitive impairment to be central to decision-making.

“What she thinks is important, we should also respect that. I believe that’s also very important, that we comply with her wishes.” (C20)

### Theme 3: Differing concerns about the future

The people with vascular cognitive impairment were mostly preoccupied with their present situation and their daily activities, which were also addressed in theme 2. Although they described their diagnosis as a negative experience or “*annoying*” (P7), they did not express much concern regarding the future and demonstrated a ‘wait-and-see attitude’.

“I’m not really worried about the [condition in the] head. And that’s because I don’t think there’s anything I can do about it anyway.” (P20)

“I’m still doing everything, I still know everything. Chores. So yes, we don’t have to talk about anything.” (P24)

A person with vascular cognitive impairment who experienced more self-efficacy had some ideas on how to handle future dependence.

“A good friend of my daughter… yes, she… she lives nearby. […] Then she can always step in, right. That’s what we… that’s what we agreed and maybe, maybe groceries need to be done if I can’t do it myself or something. I hope not, but we’ve already taken that into account. We may be getting ahead of ourselves, but it’s actually the only way.” (P26)

Caregivers were concerned about the wait-and-see attitude their relatives demonstrated. They reported that lack of awareness of the symptoms of their illness and denial were barriers to care acceptance.

“Suppose it’s going to get worse or… be at an advanced stage, then that’s going to be very difficult. […] He’s not a man who you can just say to, like: ‘Well, it’s not working, you shouldn’t be cycling anymore’. No, he’ll still take his bike, for example, because in his experience it’s all still normal and good.” (C15)

Behavioral changes, such as lack of initiative and social withdrawal, were perceived as particularly burdensome by caregivers. These behaviors affected their personal relationship and caregivers feared that it limited care acceptance.

“The change is so tough, and he has to rely on his routines. […] You mustn’t suddenly want something completely different again, when I suggest this or that. Well, no, when the weather is nice, we head outside and go somewhere… well, he doesn’t like that anymore, he doesn’t feel like doing anything.” (C13)

### Theme 4: The roles of the caregiver

Caregivers stated that they fulfilled multiple roles, such as family member, healthcare provider or even residential or financial manager. Mostly, caregivers wanted their role as family member or intimate relation to be central in their relationship.

“I also just want to be able to be close to her. […] And then it’s interesting to see, like, yes, what would be desirable from a family role’s perspective, right… I think informal care… well, you know, I’m just a daughter-in-law...” (C20)

Caregivers experienced the role of unpaid healthcare provider for their loved ones in different ways. Some said they could manage the additional time and resources spent on caregiving quite well. Caregivers often received help from a network of family and friends. On the other hand, others were sole caregivers who could not or did not want to depend on their network. Caregivers expressed the need to have time and space away from the person with vascular cognitive impairment.

“I regularly went away for a week or two. [...] So then I’m away in my own environment. Then I can invite people if I want to, or not. Because all that also diminishes, doesn’t it? Your entire social life is destroyed.” (C13)

Caregivers struggled when their caregiving role conflicted with their role as a family member or partner, as being a strict caregiver could harm the personal relationship.

“Do I have to tell him not to drive? Well, then I’ll have a war on my hands. […] Because I’ve found that very difficult, to have to decide that myself. I would’ve had to hide his keys or get rid of that car.” (C7)

The people with vascular cognitive impairment were either not aware of the role conflict caregivers experienced or did not reflect on this explicitly in the interviews. Although they expressed appreciation for their caregiver for supporting and looking after them, some indicated that the caregiver had (too) high expectations of them.

“I think I try to do my best, but that me trying my best is relative to what my wife thinks trying your best is. I think that’s a long way off.” (P7)

### Theme 5: Decisiveness from professional healthcare

“A very, very good personality. Firm. Resolute. I like that. […] I always enjoy having someone around and… well, a bit of a sparring partner.” (P42)

This person’s description accurately summarizes the ideal healthcare professional described by both people with vascular cognitive impairment and caregivers. Of note was that both groups mentioned that a healthcare professional should be resolute and decisive. People with vascular cognitive impairment mentioned that resolute professionals motivated them to pursue activities or assisted in activities that had become difficult for them.

“And it [day care] is run by two women. They also keep an eye on you, mind you. If you sit for too long: ‘Come on, we’re going outside now, let’s do this and that, clean the barn or feed the cows or something like that’. [...] And I really like that.” (P7)

Caregivers wondered whether it was possible for professionals to significantly diminish their burden.

“In fact, you should get far more [professional] care for that, so to speak. But then it would almost be like you should have someone in the house. Well, that’s to help my wife with all the things that need to be done [...] You’re always there. I mean, you always have to be there. [Later in the interview] I would like to hand that [care] over now too… I don’t know to whom...” (C29)

Caregivers valued the resolute healthcare professional, often in the person of a case manager in dementia care, who takes timely action and resolves role conflicts (theme 4).

“You can’t just say: ‘I’m breaking the cycle, I’m out of here’. You can’t. You’re in it. But someone from outside… they can see. And that was my case manager who saw it. She took action. That was very nice. I say to her: ‘I think it’s terrible that he has to go [to the nursing home]’. She said: ‘I have to, madam, otherwise I'll have to admit both of you soon’.” (C33)

## Discussion

To our knowledge, this is the first study providing in-depth insight into care needs of people with vascular cognitive impairment and their caregivers. Specifically, we found a need for more information about vascular cognitive impairment (theme 1), and the influence of vascular cognitive impairment symptoms on both concerns about the future (theme 3) and preferred professional healthcare (theme 5). In addition, we found many needs overlapping with findings from studies on dementia and stroke populations (Beunder et al., 2015; Boots et al., 2015; Granbo et al., 2019; Lindblom et al., 2020; Stiekema et al., 2020; van der Roest et al., 2007, 2009; Wolfs et al., 2010), such as the need to be valued (theme 2) and the role conflict experienced by caregivers (theme 4).

All identified themes have important healthcare implications for people with vascular cognitive impairment. Information need (theme 1) was an overarching theme (Figure 1), framing other care needs reported by our participants. Other studies have also reported the need for more information about dementia diagnoses (Boots et al., 2015; van der Roest et al., 2009; Wolfs et al., 2010), the (cognitive) consequences of cerebrovascular damage or stroke (Beunder et al., 2015; Lindblom et al., 2020; Stiekema et al., 2020), and care and treatment options. In our study, we found a discrepancy between people with vascular cognitive impairment, with low information need, and caregivers, with a need for information that was higher than was provided by healthcare professionals. Possibly, people with vascular cognitive impairment had lower information needs due to diminished awareness, or differences in acceptance and coping styles (Aminzadeh et al., 2007; Boots et al., 2015; Zwijsen et al., 2016). In theme 3 ‘Differing concerns about the future’, we discussed that coping of people with vascular cognitive impairment was characterized by a ‘wait-and-see’ attitude, a coping mechanism described earlier in people with (early-stage) dementia (de Boer et al., 2012). This could explain a relative lack of interest in information about the condition and its course. Future research should further address whether these or other factors drive low information need in people with vascular cognitive impairment (and higher information need in caregivers). An important subtheme of theme 1 was ‘No memory problem, no dementia?’. Participants equated dementia to memory complaints or commented there was no Alzheimer’s disease, therefore no dementia. We believe this subtheme is indicative of the ‘Alzheimerization’, or memory-focus, of dementia by our participants and society in general (Royall, 2003; Sachdev, 2000). Moreover, other authors have observed that patients react more positively to a diagnosis of vascular dementia than to a diagnosis of Alzheimer’s disease (Aminzadeh et al., 2007). Previous research suggests that both people with cognitive impairment and caregivers deem information about the difference between Alzheimer’s disease and dementia important (Fruijtier et al., 2019). The high information need reported by the caregivers in our study could be reflective of the limited general knowledge on vascular cognitive impairment and vascular dementia, as compared to Alzheimer’s disease (Kim et al., 2015). Therefore, better tailored information on vascular cognitive impairment and vascular dementia should be provided to the general public, as well as to people with vascular cognitive impairment and caregivers by their healthcare professionals. Increased knowledge could also improve awareness of treatment options, and thereby care utilization and self-efficacy to address unmet needs, as reported in earlier studies (Boots et al., 2015; Dam et al., 2018; Lindblom et al., 2020).

In stroke and dementia populations, a difference in insight was noted between people with cognitive impairment and caregivers on the impact of the condition on daily functioning and the future (de Boer et al., 2012; Stiekema et al., 2020). In our study, caregivers also mentioned lack of awareness of the symptoms of their illness and denial. In addition, caregivers reported behavioral changes, such as lack of initiative and social withdrawal. They discussed the challenges that these phenomena posed to care acceptance and related them to subsequent (lack of) concerns about the future (theme 3). These descriptions seem to reflect the presence of apathy, a prevalent neuropsychiatric symptom in people with cerebrovascular damage and vascular cognitive impairment (Schwertner et al., 2022; van de Schraaf et al., 2022; van der Flier et al., 2018; Wouts et al., 2020). Caregivers described changed behavior as affecting their personal relationship with the person with vascular cognitive impairment, echoing earlier research suggesting that neuropsychiatric symptoms impact the marital relationship (de Vugt et al., 2003). This stresses the importance of considering neuropsychiatric symptoms as these significantly affect caregiver burden (Dauphinot et al., 2015). Furthermore, it might be beneficial to educate caregivers about the level of acceptance and comprehension than can be expected of the person with vascular cognitive impairment (Zwijsen et al., 2016).

People with vascular cognitive impairment and caregivers had diverging views on how to deal with their prognosis and on required care after diagnosis. In line with previous literature in other populations (Stiekema et al., 2020; van der Roest et al., 2007, 2009), people with vascular cognitive impairment reported fewer instrumental care needs or demands than caregivers. They did have clear wishes regarding how they wanted to live their daily lives and how they wanted others to treat them. These wishes were reflected by theme 2 ‘Being respected as a person’. Caregivers experienced conflicting roles: as described previously, their role as a caregiver threatened their personal relationship with the person with vascular cognitive impairment (theme 4) (Granbo et al., 2019; Lindblom et al., 2020; Stiekema et al., 2020). Therefore, caregivers desired healthcare professionals who would make decisions for them at the right moment, thereby reducing perceived burden.

Participants described their preferred healthcare professional (theme 5) as resolute and decisive. In earlier studies, these characteristics were implicitly preferred by the participants. In these studies, participants stated that available care options were too “passive” (Granbo et al., 2019) or that they would feel more empowered if the healthcare professional would take the lead in (planning) the care trajectory (Lindblom et al., 2020; Wolfs et al., 2010). The decisive professional attends proactively to the care needs related to symptoms present in vascular cognitive impairment. For instance, a resolute healthcare professional could activate someone with apathy, as suggested by earlier literature on activity interventions for apathy (Brodaty & Burns, 2012). In addition, this professional could fulfil the caregiver’s need to make fewer decisions, preserving their personal relationship with the person with vascular cognitive impairment (Wolfs et al., 2010). This role was often fulfilled by a case manager in dementia care, a central contact between people with cognitive impairment, family and professional caregivers (MacNeil Vroomen et al., 2015). Other authors have emphasized that this key professional has a unique role in addressing client and caregiver needs, decreasing caregiver burden and providing continuity of care (Khanassov & Vedel, 2016; MacNeil Vroomen et al., 2015; Stiekema et al., 2020). Therefore, good case management can be pivotal for providing optimal care for people with vascular cognitive impairment and their caregivers.

At present, participants’ ideal professional care is not always available, partly due to policy decisions shifting focus to prolonged care in the community (Nederhand & Van Meerkerk, 2018). Therefore, care provision is more dependent on individual resources like strong social networks, time, money, and perseverance – one participant called it the “*ultimate elitist exercise*” (C45). The narratives in the interviews indicated that caregiving dyads with strong networks were able to cope better without professional help. Indeed, spousal caregivers desire social strong networks (Dam et al., 2018) and strong social networks have been associated with postponed institutionalization (Gaugler et al., 2000).

In today’s social climate and healthcare practice, patient autonomy is highly valued and shared decision-making is considered best practice (Elwyn et al., 2012; Peisah et al., 2013). In previous research, people with stroke and caregivers reported that they did not feel capable of having the full responsibility of their own recovery without professional support (Beunder et al., 2015; Lindblom et al., 2020; Wolfs et al., 2010). In our study, this was complemented by the expressed need for a high level of guidance or even directedness from decisive healthcare professionals. This suggests decision-making in people with vascular cognitive impairment and their caregivers requires more support and guidance from healthcare professionals (Emanuel & Emanuel, 1992; Loewy, 2005; Peisah et al., 2013). Further research is required into how healthcare professionals could guide this supported decision-making without disregarding the person’s autonomy.

A major strength of our study is the inclusion of both people with vascular cognitive impairment and caregivers, often from the same dyad, resulting in detailed understanding of both perspectives. Furthermore, we experienced a high degree of openness from all participants, assuring us of the relevance of our themes.

Our study also has limitations. Firstly, the setting of a geriatric memory clinic could have limited the diversity of the included sample. For instance, people with acute large-vessel infarctions are usually referred to a neurology rather than a geriatric clinic. By using purposeful sampling, we aimed to include participants with diverse characteristics. Secondly, due to the qualitative nature of our study design, our capacity to compare with other types of cognitive impairment is limited to comparison with the literature. Lastly, although we aimed to include people with vascular cognitive impairment without predominant other pathologies contributing to cognitive impairment, we do not have pathological or biomarker evidence to rule out other pathologies.

## Conclusions and implications

Our study highlights the importance of investigating care needs of people with cognitive impairment and family caregivers. In order to provide person-centered care, healthcare professionals should be aware of what is most important to the care recipients themselves. Our findings indicate that tailored information should be provided to people with vascular cognitive impairment and especially caregivers, as well as the general public. In this way, understanding of the prognosis and consequences of the condition and available care options will increase, thereby improving empowerment of the care recipient. In addition, neuropsychiatric symptoms in vascular cognitive impairment, particularly apathy, should be adequately addressed, as these significantly affect caregiver burden. Future research should aim to further explore differences in care needs between causes of cognitive impairment.

In this study, we investigated care needs of people with vascular cognitive impairment and their caregivers. Some of these needs are universal to all forms of cognitive impairment, others are affected by the knowledge participants had of vascular cognitive impairment or symptoms present in vascular cognitive impairment. People with vascular cognitive impairment and caregivers could benefit from tailored information, decisive healthcare professionals and more guidance to address their specific needs.

## Supplemental Material

Supplemental Material - Vascular cognitive impairment: When memory loss is not the biggest challenge Click here for additional data file. Supplemental Material for Vascular cognitive impairment: When memory loss is not the biggest challenge by Sara AJ van de Schraaf, Merel F Smit, Majon Muller, Cees MPM Hertogh, Hanneke FM Rhodius-Meester, Eefje M Sizoo in Dementia.
