# Supplementary material for: Vascular cognitive impairment: When memory loss is not the biggest challenge
Source: Dementia (London). 2023 Nov 24;23(1):152–71. doi: 10.1177/14713012231214299 (PMC10798009; doi:10.1177/14713012231214299)
Supplement: Supplemental Material - Vascular cognitive impairment: When memory loss is not the biggest challenge [file sj-pdf-1-dem-10.1177_14713012231214299.pdf]

Supplement 1. Consolidated criteria for reporting qualitative studies (COREQ) checklist

**No. Item**	**Guide questions/description**	**Details reported here and in the main text of the paper (page number)**
**Domain 1: Research team and reflexivity**		
Personal characteristics		
1. Interviewer/ facilitator	Which author/s conducted the interview or focus group?	SvdS & MS (p. 8)
2. Credentials	What were the researcher's credentials? E.g. PhD, MD	SdS: MSc<br>MS: MD<br>MM, CM, HR, ES: MD PhD
3. Occupation	What was their occupation at the time of the study?	Researcher (all), psychologist (SvdS), eldery care physician (MS, CM, ES); geriatrician (HR, MM)
4. Gender	Was the researcher male or female?	SvdS, MS, HR, MM, ES: female<br>CM: male
5. Experience and training	What experience or training did the researcher have?	SvdS completed a qualitative research training and MS has performed a qualitative interview study previously. All members of the research team have professional experience with people with vascular cognitive impairment. Moreover, CH and ES have ample experience with qualitative research.
Relationship with participants		
6. Relationship established	Was a relationship established prior to study commencement?	Four included people with vascular cognitive impairment had had previous contact with SvdS for neuropsychological evaluation. (p. 8)
7. Participant knowledge of the interviewer	What did the participants know about the researcher? e.g. personal goals, reasons for doing the research	The participants received an information leaflet with generic information about the study. Also, an introduction about the study aim and professional background of the researcher at the start of the interview (Supplement 2); no other details were provided.
8. Interviewer characteristics	What characteristics were reported about the interviewer/ facilitator? e.g. Bias, assumptions, reasons and interests in the research topic	All members of the research team have professional experience with people with vascular cognitive impairment.
**Domain 2: Study design**		
Theoretical framework		
9. Methodological orientation and theory	What methodological orientation was stated to underpin the study? e.g. grounded theory, discourse analysis, ethnography, phenomenology, content analysis	We aimed to study the lived experiences of the participants (phenomenological methodology). (p. 9)
Participant selection		
10. Sampling	How were participants selected? e.g. purposive, convenience,	We purposefully sampled individuals that could provide in-depth and complementary

	consecutive, snowball.	information, to ensure sufficient variety in the data. (p. 7)
11. Method of approach	How were participants approached? e.g. face-to-face, telephone, mail, email	People with vascular cognitive impairment were approached sending an information letter by mail and a follow-up telephone call one week later. (p. 7)
12. Sample size	How many participants were in the study?	eighteen participants (p. 7)
13. Non-participation	How many people refused to participate or dropped out? Reasons?	We contacted nineteen people with vascular cognitive impairment, and contacted a caregiver with their permission. Of thirteen people with vascular cognitive impairment, the person with vascular cognitive impairment and/or caregiver participated. (p. 7)<br>Details in this appendix not in text: Ten people with vascular cognitive impairment did not participate: five were not interested or had no time; four were not able to provide informed consent due to too severe cognitive impairment; one person had died between diagnosis and contact. Caregivers were only contacted when present and after permission of the person with vascular cognitive impairment. Therefore, numbers for non-participation of caregivers cannot be provided.
Setting		
14. Setting of data collection	Where was the data collected? e.g. home, clinic, workplace	Interviews were conducted through video call or by phone (during the Covid-19 pandemic), depending on the participant's preference. (p. 8)
15. Presence of non-participants	Was anyone else present besides the participants and researchers?	All participants were instructed to be alone during the interview, but we allowed caregivers to be present if this made the person with vascular cognitive impairment more comfortable. (p. 8)
16. Description of sample	What are the important characteristics of the sample? *e.g. demographic data, date*	Reported in Table 2.
Data collection		
17. Interview guide	Were questions, prompts, guides provided by the authors? Was it pilot tested?	Each interviewer conducted one pilot interview. The interviews were guided by a topic list, generally referring to the participants experience of received and desired care. Interview guides were revised once and are included in Supplement 2. (p. 8)
18. Repeat interviews	Were repeat interviews carried out? If yes, how many?	N.A.

19. Audio/visual recording	Did the research use audio or visual recording to collect the data?	All interviews were audiotaped and transcribed verbatim. (p. 9)
20. Field notes	Were field notes made during and/or after the interview or focus group?	Field notes were made during and after every interview. (p. 9)
21. Duration	What was the duration of the interviews or focus group?	People with vascular cognitive impairment (28-61 min) and caregivers (59-86 min). (p. 11)
22. Data saturation	Was data saturation discussed?	Data collection ended when we reached 'saturation': when we perceived richness and thickness in data, and topics discussed in later interviews fitted existing codes and themes. We expected saturation after the first fifteen interviews, and this was confirmed with three additional interviews. (p. 8)
23. Transcripts returned	Were transcripts returned to participants for comment and/or correction?	N.A.
**Domain 3: Analysis and findings**		
Data analysis		
24. Number of data coders	How many data coders coded the data?	Three: SvdS and MS coded the first seven interviews, while SvdS and ES coded later interviews. (p. 9)
25. Description of the coding tree	Did authors provide a description of the coding tree?	Supplement 3
26. Derivation of themes	Were themes identified in advance or derived from the data?	An inductive approach was used: i.e. themes were derived from the data (p. 9)
27. Software	What software, if applicable, was used to manage the data?	Atlas.ti 9 and MAXQDA 2020. (p. 9)
28. Participant checking	Did participants provide feedback on the findings?	We performed a member check; all respondents received a summary of the interview to review, two people responded (p. 9)
Reporting		
29. Quotations presented	Were participant quotations presented to illustrate the themes/findings? Was each quotation identified? e.g. participant number	Reported in the Results section. (p.11-20)
30. Data and findings consistent	Was there consistency between the data presented and the findings?	Reported in the Results section. (p.11-20)
31. Clarity of major themes	Were major themes clearly presented in the findings?	Reported in the Results section. (p.11-20)
32. Clarity of minor themes	Is there a description of diverse cases or discussion of minor themes?	Reported in the Results section. (p.11-20)

Supplement 2. Topic lists qualitative interviews

1.1 Topic list interview person with vascular cognitive impairment

- General Introduction
    a. Ask (again) if this is a good time and repeat that participant can opt-out of participation at any time.
    b. Express gratitude for participation
    c. Repeat goal of the study: to identify care needs of people with vascular cognitive impairment and informal caregivers). Explain role interviewer: scientist, but also working as a neuropsychologist/physician working with the studied population.
    d. Explain workup: interviews are audiotaped, tapes are destroyed after transcription. Quotes by the participant can appear anonymized in the final manuscript.
    e. Explain interview setting: interview questions are mostly open-ended. The interviewer will allow the participants to think about their answers, resulting in more silences than in natural conversation.

- Why did you attend the geriatric memory clinic?
    a. What were you told about the diagnosis?
    b. What were you told about available care and guidance?

- What do you notice concerning [diagnosis in wording participants]?
    a. Can you describe a typical day for you?
        i. What do you need to do and who helps you?
    b. What do you need and what is important to have good day?
    c. What you would have liked to know earlier about [diagnosis]?

- What kind of care or guidance do you receive for [diagnosis in wording participants]?
    a. What is your opinion about the received care/guidance?
    b. How did you acquire this care/guidance? What did you have to arrange?
    c. Did something change about the received care/guidance during the covid-19 pandemic?

- What kind of care or guidance would you like for [diagnosis in wording participants]?
    a. What do you need to get the desired care/guidance?

- What are important characteristics of the care/guidance you (would like to) receive?
    a. What is important for you in life?
    b. What does this care have to deliver/yield?

- Closing interview
    a. Ask about further questions, repeat goal study & thank participant.

1.2 Topic list interview family caregiver

- General Introduction
    a. Ask (again) if this is a good time and repeat that participant can opt-out of participation at any time.
    b. Express gratitude for participation
    c. Repeat goal of the study: to identify care needs of people with vascular cognitive impairment and informal caregivers). Explain role interviewer: scientist, but also working as a neuropsychologist/physician working with the studied population.
    d. Explain workup: interviews are audiotaped, tapes are destroyed after transcription. Quotes by the participant can appear anonymized in the final manuscript.
    e. Explain interview setting: interview questions are mostly open-ended. The interviewer will allow the participants to think about their answers, resulting in more silences than in natural conversation.

- Why did you attend the geriatric memory clinic?
    a. What were you told about the diagnosis?
    b. What were you told about available care and guidance?

- What do you notice concerning [diagnosis in wording participants]?
    a. If things are challenging, how do you solve this? What do you need to cope with these challenges?
    b. What you would have liked to know earlier about [diagnosis]?

- What kind of care or guidance do you receive for [diagnosis in wording participants]?
    a. What is your opinion about the received care/guidance?
    b. How did you acquire this care/guidance? What did you have to arrange?
    c. Did something change about the received care/guidance during the covid-19 pandemic?

- What kind of care or guidance would you like for [diagnosis in wording participants]?
    a. What do you need to get the desired care/guidance?
    b. What kind or care/guidance would you like for yourself as a caregiver?

- What are important characteristics of the care/guidance you (would like to) receive?
    a. What is important for you concerning the life of your loved one (the person with vascular cognitive impairment?
    b. What does this care have to deliver/yield?

- Closing interview
    a. Ask about further questions, repeat goal study & thank participant.

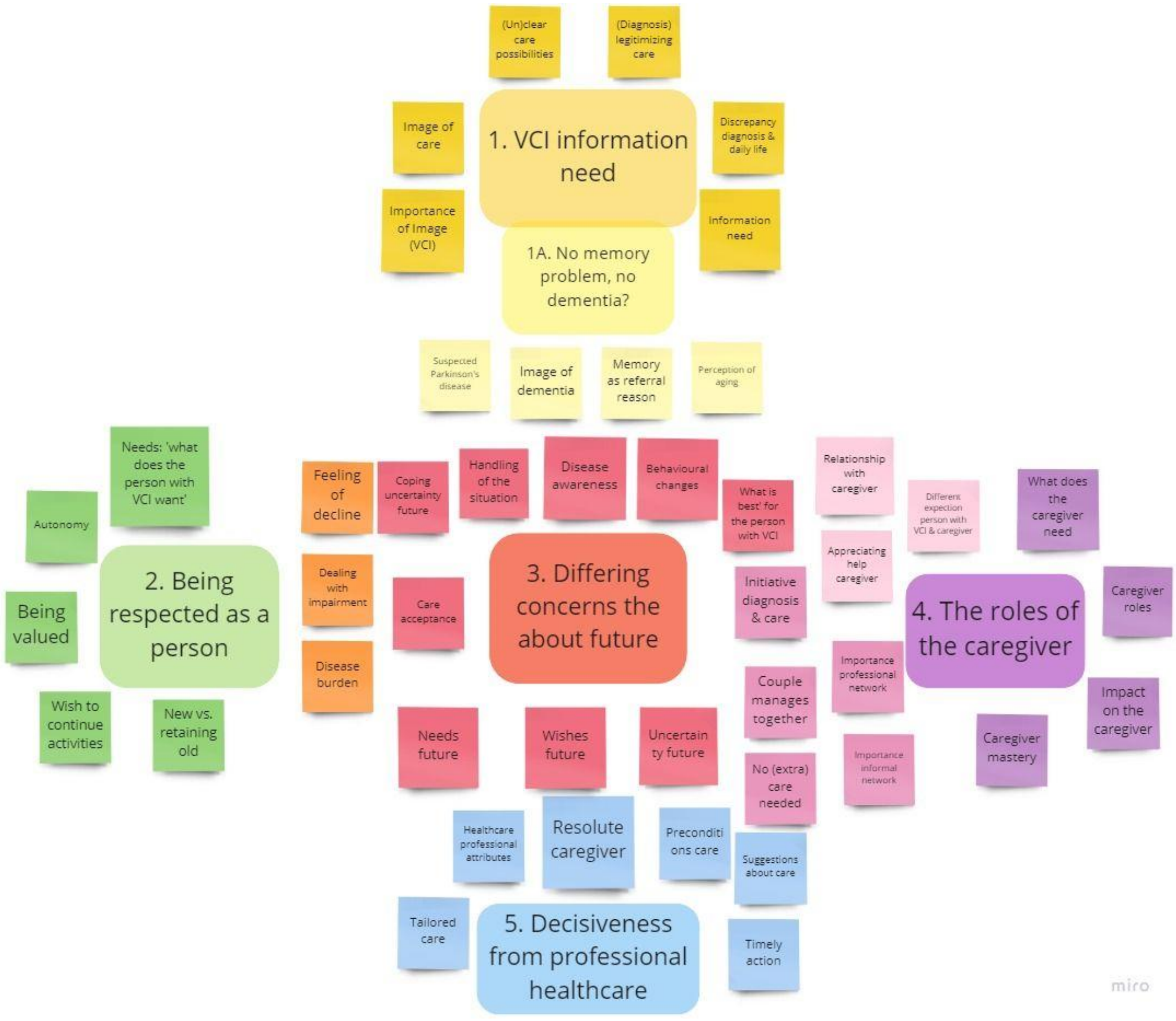

Supplement 3. Coding cloud of the themes in care needs of people with vascular cognitive impairment and their caregivers.
